# Solid serous microcystic adenoma of the pancreas

**DOI:** 10.1186/1477-7819-5-26

**Published:** 2007-03-05

**Authors:** Jordan R Stern, Wendy L Frankel, E Christopher Ellison, Mark Bloomston

**Affiliations:** 1 Department of Surgery, The Ohio State University, 410 W 10th Ave, N924 Doan, Columbus OH 43210, USA; 2 Department of Pathology, The Ohio State University, Columbus OH 43210, USA

## Abstract

**Background:**

Cystic neoplasms of the pancreas are less common than solid tumors, and portend a better prognosis. They can be divided into serous and mucinous subtypes, with the former behaving less aggressively and generally considered benign. Of the serous neoplasms, serous microcystic adenoma is the most common. An extremely rare solid variant of serous microcystic adenoma lacking secretory capability has been described. Herein, we present the fourth described case of this solid variant and review the literature.

**Case presentation:**

We present a case of a 62 year-old man with a history of abdominal pain, who on CT scan was found to have a solid mass at the junction of the head and body of the pancreas. The patient was offered resection for diagnosis and treatment, and subsequently underwent distal pancreatectomy and splenectomy. Based on gross pathology, histology and immunohistochemistry, the mass was determined to be a solid serous microcystic adenoma.

**Conclusion:**

Solid serous microcystic adenoma shows similar histologic and immunohistologic features to its classic cystic counterpart, but lacks any secretory functionality. It appears to behave in a benign manner, and as such, surgical resection is curative for patients with this tumor. Furthermore, until more cases of solid SMA are identified to further elucidate its natural history and improve the reliability of preoperative diagnosis, surgical resection of this solid pancreatic tumor should be considered standard therapy in order to exclude malignancy.

## Background

Tumors of the pancreas generally portend a poor prognosis, the majority of these being highly malignant ductal adenocarcinomas [1]. Much less common are the pancreatic cystic neoplasms, which include serous and mucinous subtypes, among others. Together these comprise only 1–2% of all pancreatic exocrine tumors [2]. Architecturally, the serous cystic tumors are comprised of cuboidal cells arising from the pancreatic ductal epithelium, and having clear, glycogen-rich cytoplasm with small, central nuclei [1]. An important clinical distinction is that the serous neoplasms tend to be benign, whereas their mucin producing counterparts may go on to malignancy [3]. Exceptions to this include the few cases of serous adenocarcinoma that have been reported [4-6]; these serous adenocarcinomas can show both direct spread [7] and distant metastases [6]. Serous pancreatic neoplasms can be identified by the pattern of histochemical (periodic acid-schiff [PAS] and mucin) and immunohistochemical staining. They show positive staining for PAS that is diastase sensitive. Typically, they stain positive for various cytokeratins, neuron-specific enolase (NSE), α-inhibin, MUC-1 and MUC-6 [1,8], and negative for α-smooth muscle actin, S-100 protein, carcinoembryonic antigen (CEA), chromogranin and synaptophysin [8].

Serous microcystic cystadenoma (SMA) is the most commonly encountered of the serous cystic neoplasms. On gross examination, SMA typically is a well circumscribed, round to ovoid lesion with a spongy consistency [7]. Often there is a central fibrous scar in a stellate pattern [3]. Surrounding the scar are innumerable small cysts containing a thin, clear fluid [3]. These lesions are most often single, although multifocal SMA has been reported [2]. An oligocystic form has also been described which, in contrast to SMA, contains only a few large cystic spaces [9].

A rare solid variant of SMA has been described in three previous cases [1,10,11]. Although the cytological and architectural features are largely the same, these tumors are distinguished from the classic SMA by their lack of fluid filled cysts, which gives them a more compact structure [10]. The first reported case of solid SMA was discovered in a 70 year-old female with a history of diffuse abdominal pain. She was found to have a 4 cm well-defined, solid mass in the tail of the pancreas on CT, which was then resected by distal pancreatectomy. The second case was a 2.5 cm solid tumor in the pancreatic head of a 50 year-old male patient, which was well circumscribed and separate from a concurrent pancreatic ductal adenocarcinoma. The third, a 4 cm solid mass at the junction of the pancreatic head and body, was found incidentally on workup for an unrelated problem in a 69 year-old male. Both of these more recent cases were resected by pylorus-preserving pancreaticoduodenectomy. Herein we report a fourth case of solid serous microcystic adenoma of the pancreas.

## Case presentation

### Clinical course

**Figure 1:**
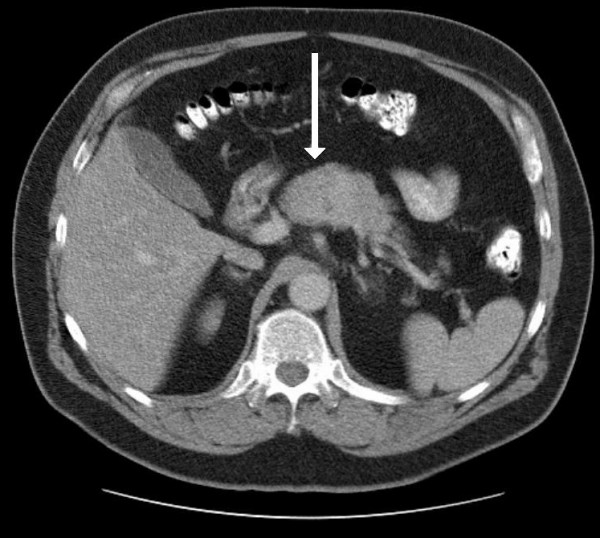
CT scan of the abdomen with contrast. A heterogeneously enhancing, solid mass can be identified at the junction of the head and body of the pancreas (arrow).

A 62 year-old man with a several month history of abdominal pain was referred to The Ohio State University Medical Center in June, 2006. His past medical history was significant for hypertension, arthritis and chronic back pain. On computed tomography scan he was found to have a heterogeneously enhancing solid mass at the junction of the head and body of the pancreas (Figure 1). Based on initial imaging, the differential diagnosis included pancreatic ductal adenocarcinoma, pancreatic endocrine tumor, solid pseudopapillary tumor, and metastatic carcinoma.

The patient was offered resection for diagnosis and definitive therapy. After undergoing diagnostic laparoscopy to rule out carcinomatosis in the peritoneal cavity, a distal pancreatectomy with splenectomy was completed.

The patient tolerated the procedure well and was discharged after an uneventful hospital stay.

### Pathological findings

**Figure 2:**
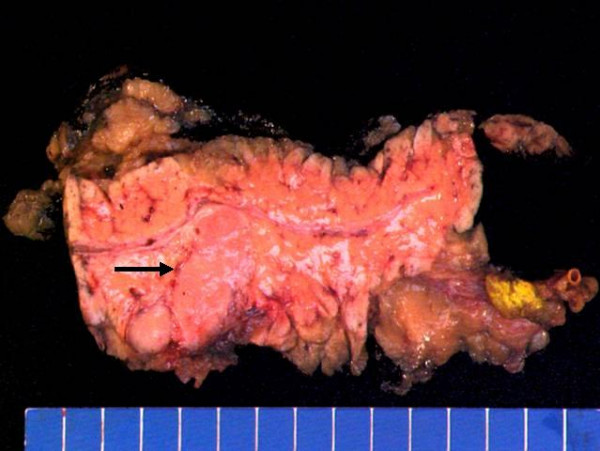
Distal pancreas, gross specimen. An ovoid, well circumscribed, solid mass measuring 4.2 × 2.5 × 2.0 cm is seen adjacent to the pancreatic duct and extending to the periphery of the specimen (arrow).

**Figure 3:**
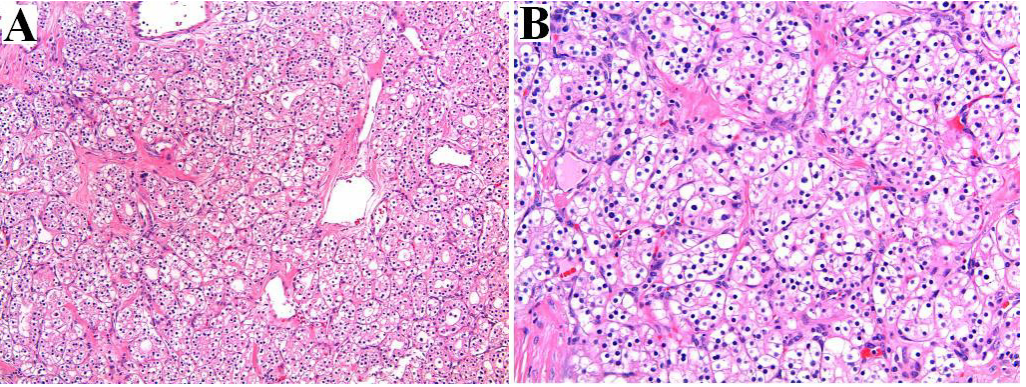
Hematoxylin and eosin stain. **(A) **Low-power magnification (10×) shows irregular trabeculae and glandular structures, separated by hypocellular fibrous bands. Note the absence of cystic spaces within the glands. **(B) **Higher magnification (40×) demonstrates a homogenous population of polygonal cells with clear cytoplasm and small, central, round nuclei.

**Figure 4:**
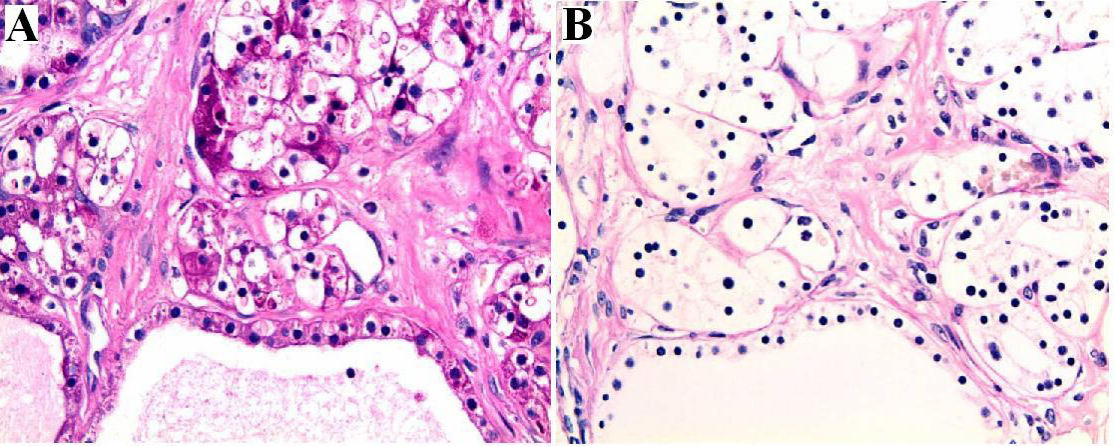
Periodic Acid-Schiff (PAS) stain. **(A) **Strong reaction with PAS is evident within the cytoplasm of the polygonal cells. **(B) **Staining disappears in the presence of diastase.

Gross examination of the resected specimen demonstrated a 4.2 × 2.5 × 2.0 cm solid, well circumscribed, non-encapsulated mass adjacent to the pancreatic duct (Figure 2). The cut surface of the tumor contained thick fibrous bands without hemorrhage or necrosis. The surrounding pancreatic parenchyma was noted to be grossly unremarkable, and surgical margins were negative. On hematoxylin and eosin stain, the architecture of the mass was solid with cells arranged in nests, acini and trabeculae, separated by thick, hypocellular fibrous bands (Figure 3A). Rare small cystic areas were seen microscopically (less than 1% of the tumor). The tumor was homogenous and consisted of polygonal cells with centrally placed small, round to ovoid nuclei (Figure 3B). No mitoses or cellular atypia were noted. The cells contained abundant clear cytoplasm which was PAS positive and diastase sensitive suggesting the presence of glycogen (Figure 4). No vascular or perineural invasion was seen.

Mucicarmine stain was negative. Immunohistochemistry showed positive staining for cytokeratin 7 (CK7), cytokeratin 19 (CK19), neuron-specific enolase (NSE), α-inhibin and calponin; and negative staining for vimentin, α1-antitrypsin (A1AT), synaptophysin, carcinoembryonic antigen (CEA), renal cell carcinoma stain (RCC), CD10, S-100 protein, and smooth muscle actin. These findings support the final diagnosis of solid microcystic adenoma.

## Discussion

For a solid, clear cell tumor in the pancreas, the differential diagnosis includes neuroendocrine tumors, solid pseudopapillary tumor, ductal adenocarcinoma, acinar cell carcinoma, solid serous microcystic adenoma, and metastatic clear cell carcinoma of renal, myoepithelial or other origin. A diagnosis of solid SMA was made in this case after considering gross pathology, histology and immunostaining characteristics.

The previous cases of solid SMA were all well-circumscribed, spherical masses ranging in size from 2.5–4.0 cm. Our specimen shared these characteristics and was approximately the same size, with a maximal dimension of 4.2 cm. Histologically our case is virtually identical. All four have had the same general solid architecture: thick fibrous bands separating glandular structures composed of clear, cuboidal cells with central nuclei and abundant glycogen.

Further evidence supporting the diagnosis came from immunohistochemical studies. Positive staining for CK7, CK19 and NSE is consistent with the other solid SMA cases, and these markers are generally present in serous microcystic adenomas [10]. The absence of chromogranin and synaptophysin staining argues against a neuroendocrine tumor. Solid pseudopapillary tumor is also unlikely given negative staining for vimentin and A1AT and diffuse strong staining with several cytokeratins.

Renal and myoepithelial cell carcinomas can metastasize to the pancreas, and may be difficult to distinguish from a primary pancreatic cancer [12]. This tumor showed negative staining for RCC, vimentin, CD10 and smooth muscle actin, which does not support a metastatic lesion with renal or myoepithelial cell origin. Interestingly though, staining for calponin was positive, something not evaluated in the previous cases of SMA.

## Conclusion

Solid tumors of the pancreas are typically associated with malignancy, whereas cystic tumors more often tend to be benign [11]. Given the histologic and immunohistochemical characteristics of solid serous microcystic adenoma, it is likely more related to the cystic tumors than to the solid ones, as its gross pathology might suggest. Clinically this holds true as well, as the three previous cases have all been considered benign lesions without evidence of local spread or metastasis. Given the benign nature of solid SMA, surgical resection most likely represents definitive treatment and cure. In addition, resection is indicated due to the difficulty in diagnosing solid SMA preoperatively. Most solid tumors of the pancreas should be resected due to their high malignant potential, and since a diagnosis of solid SMA will likely not be made until histologic and immunohistochemical analyses are completed, it should be treated as a potentially malignant lesion as well. Thus, until more cases of solid SMA are identified to further elucidate its natural history and improve the reliability of preoperative diagnosis, surgical resection should still be considered standard therapy in suitable patients to exclude malignancy.

## Competing interests

The author(s) declare that they have no competing interests.

## Authors' contributions

**JRS**: data collection, literature review and manuscript preparation

**WLF**: critical review

**ECE**: critical review

**MB**: data collection and critical review
